# Slittable sheath supported right ventricular pacing lead implantation in persistent left superior vena cava with absent right superior vena cava: a case report

**DOI:** 10.1186/s13256-023-04073-y

**Published:** 2023-08-14

**Authors:** Jiří Plášek, Jiří Vrtal, David Šipula, Tomáš Grézl, Jan Václavík

**Affiliations:** 1 Department of Internal Medicine and Cardiology, University Hospital Ostrava, 17. Listopadu 1790/5, 708 52, Ostrava, Czech Republic; 2 Department of Cardiology, Institute for Clinical and Experimental Medicine, Vídeňská 1958, 140 21, Prague, Czech Republic; 3 Centre for Research on Internal Medicine and Cardiovascular Diseases, Faculty of Medicine, University of Ostrava, Syllabova 19, 703 00, Ostrava, Czech Republic

**Keywords:** Cardiac pacing, Persistent left superior vena cava, PLSVC, Right superior vena cava, RSVC, Slittable sheath

## Abstract

**Background:**

Persistent left superior vena cava (PLSVC) is the most common variant of systemic venous drainage. In the absence of the right superior vena cava (RSVC), implantation of a right ventricular pacing lead may be challenging. Therefore specific implantation techniques and experiences in PLSVC are worth reporting.

**Case presentation:**

We present a case report of a 90-year-old Caucasian female patient with PLSVC during single chamber pacemaker implantation due to the third-degree atrioventricular block. With common implantation techniques, we did not even reach the right ventricle. Therefore slittable CPS Direct ™ Universal sheath was employed to overcome the acute angle from PLSVC to tricuspid valve and ensure more fixation stability for longer 100-cm right ventricular lead placement.

**Conclusion:**

This case demonstrates safe implantation of 100-cm long right ventricular bipolar active fixation pacing lead using common slittable CPS Direct ™ Universal sheath after failed attempts with „C“ and „J“ stylet shaped electrode. This sheath provides different angle towards tricuspid valve and more fixation stability in patient with PLSVC and absent connection to right atrium.

## Background

Persistent left superior vena cava (PLSVC) is the most common variant of systemic venous drainage. In the absence of the right superior vena cava (RSVC), implantation of a right ventricular pacing lead may be challenging. Therefore specific implantation techniques and experiences in PLSVC are worth reporting.

## Case presentation

We present a case report of a 90-year-old Caucasian female patient with PLSVC during single chamber pacemaker implantation due to the third-degree atrioventricular block. At initial evaluation the patient was hypertensive (155/95 mmHg), with decreased peripheral oxygen saturation of 94% on room air, mild tachypnoea of 25 breath per minute, basal inspiratory crackles, and otherwise normal physical examination. The patient was admitted to our department due to syncope with no prior history of antiarrhythmic drug treatment, recent hyperkalaemia, or Lyme disease.

**Fig. 1:**
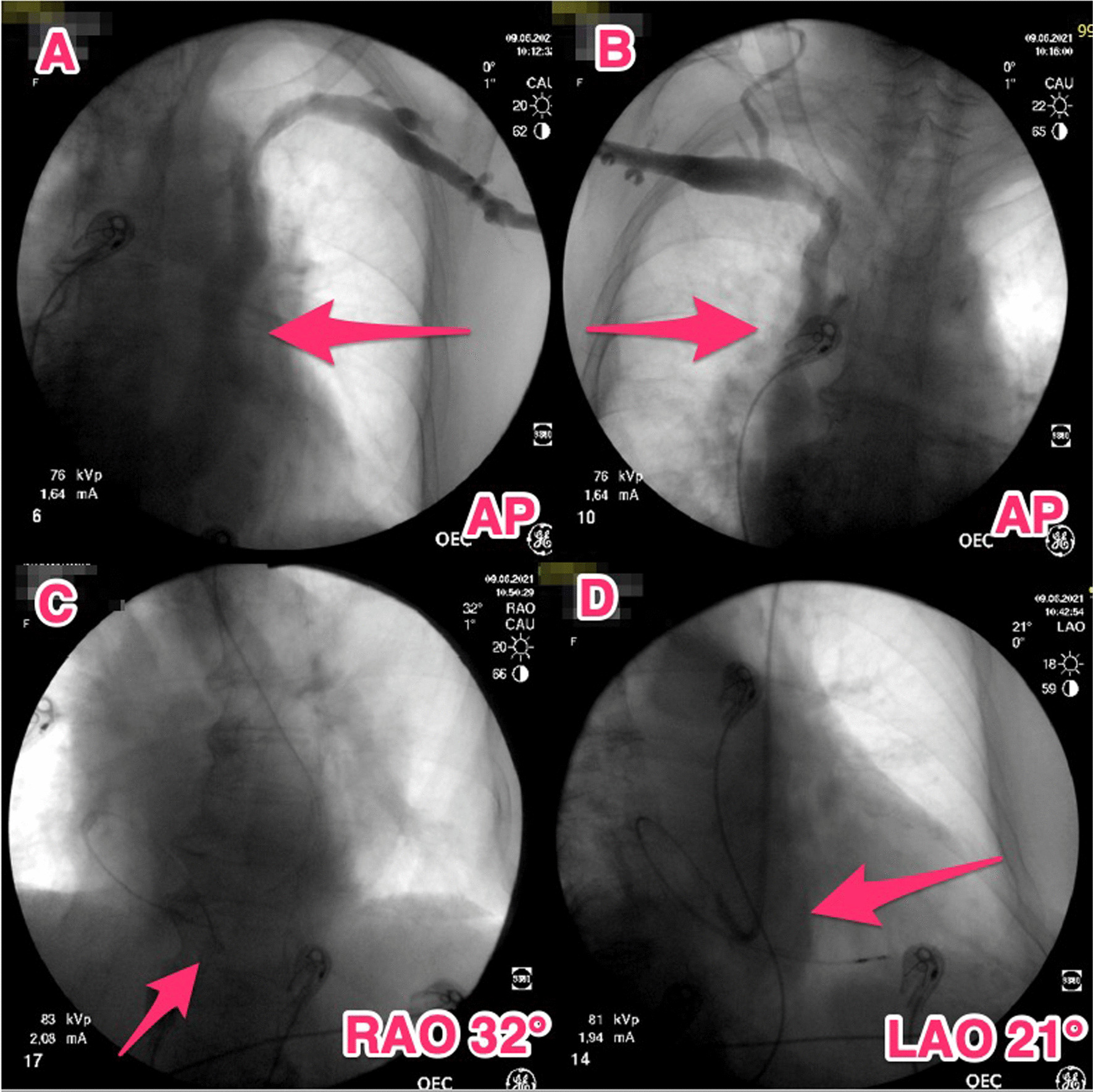
Chest X-ray. **A** posteroanterior projection; contrast visualization of persistent left superior vena cava (arrow); **B** posteroanterior projection; contrast visualization of absent right superior vena cava, subclavian vein draining into the azygous vein**;** right anterior oblique projection of cardiac pacing lead (arrow); **D** left anterior oblique projection of cardiac pacing lead in „alpha “ loop contouring the shape of coronary sinus

**Fig. 2:**
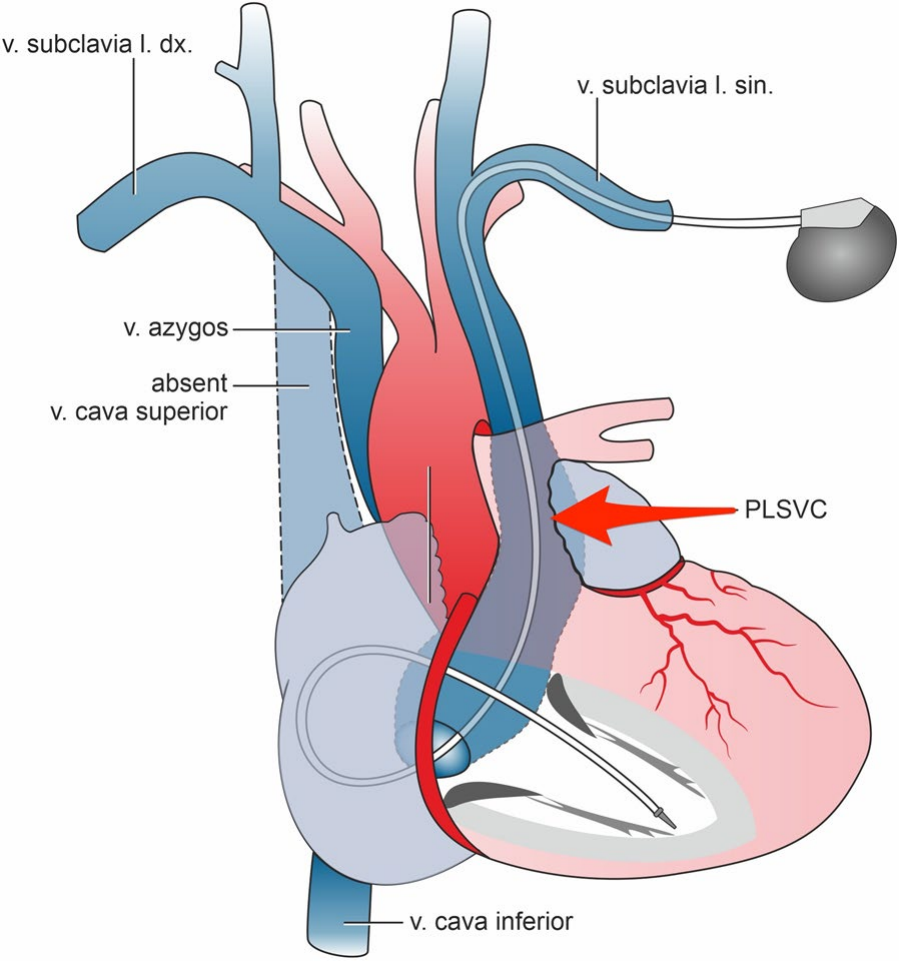
Illustration depicting the route of right ventricular pacing lead through persistent left superior vena cava

After entering the venous system via the left subclavian vein, an unusual guidewire course was observed. A wide vein circumventing the left heart silhouette draining into the right atrium was detected on phlebography (Fig. 1A). Contralateral venography showed absent RSVC. Right subclavian vein drained into the azygous vein; visible vein irrespective of the right atrial shadow borders (Fig. 1B). Therefore, implantation had to proceed from the left side. A 65-cm bipolar active fixation pacing lead (Tendril STS 2088, Abbott, Minneapolis, USA), with „C “and later „J “ loop stylet shaping, was employed. Nevertheless, we could not place the electrode through the tricuspid annulus, probably due both to the sharp angle from the coronary sinus and tricuspid regurgitation. Thus, after failed attempts slittable curved outer guide 10F catheter (CPS Direct™ Universal, Abbott, Minneapolis, USA) for coronary sinus cannulation with 100-cm bipolar active fixation pacing lead (Tendril STS 2088, Abbott, Minneapolis, USA) was used, enabling an appropriate approach to the tricuspid valve and support for the right ventricular lead placement (Fig. 1C, D). CPS catheter was guided via 0.032" guidewire to the right atrium. Thereafter 100-cm pacing lead was easily advanced with counterclockwise rotation of the sheath through the tricuspid valve to the right ventricular apex (Fig. 1C, D). For the advancement of the lead mostly right anterior oblique projection was used since the course of both lead and the sheath were easily visible. Thereafter left anterior oblique projection was employed for the fixation of the lead. Final alpha shape of the ventricular lead retained due to the lead implantation path, see also Fig. 2. Echocardiography examination revealed dilated right atrium (apical four chamber 45 × 60 mm), borderline right ventricular diastolic diameter (parasternal long axis 28 mm, apical four chamber view 43 mm), dilated coronary sinus and second-degree tricuspid regurgitation.

## Discussion

We decided for single chamber (VVI) and not dual chamber (DDD) pacemaker for several reasons. First the patient was of advanced age, and it was shown in the UKPACE trial that the pacing mode (VVI vs. DDD) does not influence the rate of death from all causes in the first 5 years or incidence of cardiovascular events during the first three years after implantation [1]. Secondly, the anatomy was quite challenging, even though atrial lead would have been probably easier to place than the ventricular lead but in non-standard position. And that lead us to the third reason, the safety of atrial lead implantation in PLSVC scenario. Perforation of the atrial lead is quite a rare phenomenon with incidence ranging from 0.3 to 0.5%, in PLSVC patients however the incidence is up to7.6% [2].

At 1-year follow-up, the patient was doing well, with no heart failure, presyncope, or syncope symptoms. On echocardiography, paradoxical ventricular septal motion was present, but the left ventricular ejection fraction remained normal (55%).

Of note, if we would not be successful in implanting endocardial lead, a leadless pacemaker would have been an option. The primary disadvantage of a leadless pacemaker is the large diameter of the delivery sheath (27-French outer diameter), which may be complicated to advance, and the price of the whole system. PLSVC is the most common variant of systemic venous drainage with an incidence of 0.3 to 0.5%; in 20% of patients, RSVC is absent [3]. PLSVC typically drains into the coronary sinus, dilatation of which may be the first hint [3]. In the minority of the cases, it drains into the left atrium via the unroofed coronary sinus [3]. Although PLSVC is in the majority of cases hemodynamically insignificant and found incidentally, it may pose a trouble when placing venous lines or cardiac implantable devices (CIEDs). Therefore, mostly right-sided approach is recommended [4]. In patients with absent RSVC, the placement of a cardiac device through PLSVC is challenging.

The implantation success depends on both individual patient anatomy and operator experience. However, few significant obstacles must be expected in this scenario: (1) greater distance to the right ventricle demanding longer electrode (2) acute angle from the PLSVC/coronary sinus to the tricuspid valve (3) inadequate wall support of the heart chambers given the dilated coronary sinus. While predominantly adequate stylet shaping [4, 5] of the ventricular pacing lead may overcome the complex anatomy of the PLSVC, in specific cases, longer electrode and slittable sheath prove helpful. To the best of our knowledge, only one similar case is described using Worley CSG™ sheath in advancing pacing electrode safely to the right ventricle in PLSVC [6]. In our opinion any curved (115–135°) long sheath may be helpful. However, a peel away or slittable sheath portends certain advantages due to remaining electrode stability after removing the sheath.

## Conclusion

This case demonstrates safe implantation of 100-cm long right ventricular bipolar active fixation pacing lead using more common slittable CPS Direct™ Universal sheath in PLSVC patient with absent RSPVC after failed attempts with „C “and „J “ stylet shaped electrode.

## Key teaching points

- Adequate „C“and later „J“ loop stylet shaping may overcome the complex anatomy of persistent left superior vena cava (PLSVC)
- Longer pacing lead (65–100 cm) is desirable to reach right ventricle in PLSVC.
- In specific cases long slittable/peelable sheath proved helpful

## Data Availability

Not applicable.

## Abbreviations

CIEDS: Cardiac implantable devices
ICD: Implantable cardioverter defibrillator
DDD: Dual chamber pacemaker with both inhibition and triggered reaction
VVI: Single chamber “on-demand” pacemaker regime
PLSVC: Persistent left superior vena cava
RSVC: Right superior vena cava

## Data availability

The data that support the findings of this study are available from the corresponding author upon reasonable request and with compliance to the General Data Protection Regulation.
